# Supplementary material for: Effects of dry cupping on exercise, autonomic activity and sleep in baseball players during preseason and in-season conditioning
Source: PLoS One. 2025 Feb 25;20(2):e0319479. doi: 10.1371/journal.pone.0319479 (PMC11856306; doi:10.1371/journal.pone.0319479)
Supplement: Attachment — Submitted filename: Rebuttal letter-PLosOne0123.doc [file pone.0319479.s001.doc]

**Sites of Revision and Reply to the Comments of the Reviewers**

**(Manuscript Number: PONE-D-24-57248)**

Thank you for your constructive and positive review of our manuscript entitled “**Effects of dry cupping on exercise, autonomic activity and sleep in baseball players during preseason and in-season conditioning**”. Our responses are included in the following pages. All the changes (included text and references) in the revised manuscript made in response to these comments are clearly indicated with red highlights. We hope that the revisions are in order.

**Responses to Reviewers’ Comments**

### **Reviewer : 1**

### 1. Mention sample size calculations in method section instead of analysis section.

**Response:** We sincerely appreciate the reviewer’s insightful comment. In response to the suggestion, we have revised the manuscript accordingly by relocating the description of the sample size calculations to the first paragraph of the Methods section (Lines 93–99 of the revised version). This adjustment ensures better alignment with the section's overall structure and enhances the clarity of the presentation.

**Changes in the manuscript**:

The adequacy of the sample size for this study was evaluated using G-Power software (version 3.1.9.4, Heinrich-Heine-Universität Düsseldorf, Germany). An a priori power analysis for repeated measures analysis of variance (RM-ANOVA) was conducted, with the following parameters: an effect size of .60 [22], a statistical power of .80, and an alpha level of .05. The analysis indicated a minimum required sample size of 24 participants. Thus, the final sample of 40 participants exceeded the minimum requirement, supporting the statistical validity of the study.

2. Write the name of statistical tests that were applied.

**Response:** We sincerely thank the reviewer for their valuable comments. The statistical test applied in this study was **repeated measures analysis of variance (RM-ANOVA)**. This has been described in the estimation of sample size (Line 95) and in the opening line of the Statistical Analysis section (Line 215).

3. Mention how qualitative and quantitative measures were calculated.

**Response:** Thank you for your thoughtfulness bringing this to our attention. The analysis of questionnaire scales and physiological parameters in this study comprised exclusively quantitative measurements. No qualitative data were collected, as this study did not include qualitative interviews or other qualitative assessment methods. The quantitative analysis methods of this study are described in the statistical analysis section (lines 215-225).

**Changes in the manuscript**:

Qualitative measures were analyzed using RM-ANOVA to evaluate the pretest and posttest data for both the PSC period (week 1 vs. week 4) and ISC period (week 4 vs. week 8), focusing on the time effect and group × time interactions across various parameters in the cupping and sham groups…………………..This helped ensure that the findings were interpreted not only in terms of statistical significance but also in their practical implications for athletic performance and recovery.

4. In tables, mention which tests are applied in legend and in columns, mention if mean/median/IQR/SD were calculated, many readers may not understand without respective headings.

**Response:** Thank you for your insightful comment. We have added the statistical tests applied to the data in the captions of Tables 1–4 and Figures 3 and 4. Additionally, we have explicitly stated that the data are presented as mean ± standard error of the mean (SEM). These revisions were implemented to enhance clarity and ensure that readers can readily interpret the data.

**Changes in the manuscript**:

1). **Add the following to the footnotes of Tables 1 to 4:** Data are presented as mean ± standard error of the mean (SEM). Statistical analysis was performed using RM-ANOVA.

2). **Caption for Fig 3:** The rate of LF recovery was significantly higher in the cupping group compared to the sham group after 4 weeks of intervention, as determined by RM-ANOVA, which showed a significant group × trial × time interaction effect (P = .013).

3). **Caption for Fig 4:** Effects of cupping on postexercise LF% recovery after strenuous exercise were analyzed using RM-ANOVA.

5. Table 3 needs formatting. If possible, present this page in landscape.

**Response:** Thank you for your suggestion. In response, we have reformatted Table 3 and presented it in landscape orientation as requested. The updated table has been uploaded as supplementary information for better readability.

6. Line 314 - 318: Cite references for these theories.

**Response:** The theories mentioned in this section are primarily derived from a review article (Reference 33, cited in line 337 and listed in lines 563-565). This article provides a systematic review of the mechanisms of cupping therapy as proposed by recent scholars and summarizes the various mechanisms discussed throughout the text.

**Reference 33:**

Al-Bedah AMN, et al. The medical perspective of cupping therapy: Effects and mechanisms of action. J Tradit Complement Med. 2018; 9(2): 90-97. doi: 10.1016/j.jtcme.2018.02.003

**Reviewer: 2**

**Abstract**

1. The abstract provides a clear summary of the study’s objectives, methods, results, and conclusions. However, it could benefit from slightly tighter phrasing to improve readability. For example: Instead of “Cupping during PSC significantly improved daytime dysfunction scores on the Pittsburgh sleep quality index,” consider “Cupping during PSC notably enhanced daytime function, as measured by the Pittsburgh Sleep Quality Index.”

**Response:** We are very grateful for the reviewer’s comments. In response, we have revised the sentence in lines 38-39.

**Changes in the manuscript**:

Cupping during PSC notably enhanced daytime function, as measured by the Pittsburgh Sleep Quality Index.

**Introduction**

2. I suggest to add a brief mention of other non-pharmacological recovery methods for comparison, enhancing the context for cupping.

**Response:** Thank you for your valuable suggestion. We agree that providing a brief mention of other non-pharmacological recovery methods would enhance the context for cupping and offer a comparative perspective. In response, we have added a brief discussion of relevant non-pharmacological recovery approaches to lines 60-63 of the revised manuscript. This addition serves to highlight the broader landscape of recovery strategies and situates cupping within this context.

**Changes in the manuscript**:

It is common for athletes to employ non-pharmacological interventions, such as alternating hot and cold-water therapy, cryotherapy, compression garments, electrical stimulation, and massage, to enhance recovery from training-induced fatigue [Reference 10]. However, these methods may not be as economical or convenient as cupping.

**Newly added reference:**

[10] Barnett A. Using recovery modalities between training sessions in elite athletes. Sports Med. 2006; 36(9): 781-796. doi.org/10.2165/00007256-200636090-00005

3. The research question is clearly articulated. However, the hypothesis could be more explicitly stated.

**Response:** Thank you for your valuable feedback. In response, we have explicitly stated our hypothesis in the Introduction (Lines 77-81 of the revised version) to clarify the expected outcomes of the study. We hope this revision addresses your comment effectively.

**Changes in the manuscript**:

We hypothesized that regular dry cupping applied to the upper body muscles (trapezius and deltoids), commonly used during pitching and batting, would lead to improvements in subjective perceptions, such as sleep quality and upper limb and shoulder function, as well as objective physiological responses related to ANS activity during both the PSC and ISC periods.

**Methods**

4. It would help to specify whether the randomization process was blinded to participants and researchers.

**Response:** Thank you for your insightful comment. In response, we clarify that the randomization process was single-blinded. The participants were blinded to their group allocation to minimize potential bias in subjective assessments. However, due to the necessity of applying specific cupping pressures accurately, the researchers were aware of each participant’s assigned group. This approach ensured the correct implementation of the intervention while maintaining blinding on the participants' side to enhance the study's internal validity. We revised the manuscript accordingly to provide this clarification (Lines 109-112).

**Changes in the manuscript**:

To minimize potential bias in subjective assessments, the randomization process was single-blinded, meaning that participants were unaware of their group allocation. However, researchers were informed of the assignments to ensure the accurate application of the intervention, particularly the cupping pressure.

5. A flow diagram could make the experimental design and participant flow more accessible.

**Response:** Thank you for your constructive suggestion. In response, we have revised the experimental design and participant flow in the original **Figure 1** to enhance clarity and accessibility. A revised flow diagram has been created and included in the updated manuscript to visually represent the study design and participant allocation process, thereby improving the overall readability and comprehensibility of the methodology.

**Changes in the manuscript**:

6. HRV data collection and analysis are described rigorously. However, briefly explaining why LF and LF/HF were prioritized over other HRV indices would provide clarity.

**Response:** Thank you for your insightful comment. The prioritization of LF and the LF/HF ratio was based on their established relevance in assessing autonomic nervous system function during exercise, as they are widely used in the literature. We chose these indices for their robust applicability, while also acknowledging the importance of other HRV parameters, such as HF, in providing a comprehensive analysis. Further details on this rationale are provided in lines 194-202 of the manuscript.

**Changes in the manuscript**:

The LF component primarily reflects the combined influence of sympathetic and parasympathetic activity, with a predominant association to sympathetic modulation. The LF/HF ratio is commonly used to assess the balance between sympathetic and parasympathetic activity. However, the interpretation of the LF/HF ratio is complex and may not accurately reflect sympathovagal balance due to various influencing factors. In our study, we prioritized LF and LF/HF ratio over other HRV indices based on their widespread application in assessing autonomic function during exercise. We acknowledge the complexities in interpreting these indices and recognize the importance of considering additional HRV parameters, such as HF, to provide a more comprehensive evaluation of autonomic function.

7. Include post hoc testing methods for significant findings.

**Response:** Thank you for your valuable feedback. In our study, we employed repeated measures analysis of variance (RM-ANOVA) to assess the effects of cupping on sleep quality during the PSC and ISC periods. Given that our analysis involved only two groups—cupping and sham—post hoc tests were not conducted. This approach aligns with standard statistical practices, as post hoc analyses are typically reserved for situations involving multiple group comparisons to identify specific group differences.

8. Highlighting clinically meaningful outcomes, in addition to statistical significance, could add value.

**Response:** Thank you for your valuable suggestion. We acknowledge the importance of considering clinically meaningful outcomes alongside statistical significance to enhance the practical applicability of our findings. In response, we will incorporate a discussion on the clinical relevance of the observed changes in key parameters, such as peak power, exercise duration, and HRV activity. Specifically, we will evaluate whether the magnitude of changes observed in both the cupping and sham groups aligns with established thresholds for clinically meaningful improvements reported in the literature (Lines 221-225). Furthermore, we will contextualize our findings by discussing their potential implications for athletic performance and recovery strategies (See discussion section).

We appreciate your insightful feedback and believe that these additions will provide a more comprehensive interpretation of our results.

**Changes in the manuscript**:

In addition to assessing statistical significance, the clinical relevance of the observed changes was considered by evaluating whether the magnitude of improvements aligns with established thresholds reported in the literature. This helped ensure that the findings were interpreted not only in terms of statistical significance but also in their practical implications for athletic performance and recovery.

**Discussion**

9. Consider integrating more mechanistic insights into why sleep improvements were limited to PSC.

**Response:** Thank you for your valuable suggestion. In response, we have integrated more mechanistic insights into the discussion to explain why sleep improvements were observed primarily during the PSC period (Lines 412-423). We believe this addition provides a clearer understanding of the differential effects of cupping across the two conditioning periods.

**Changes in the manuscript**:

1). Lines 412-416: The observed sleep improvements during PSC may be attributed to cupping’s ability to promote relaxation and reduce muscle tension, which may contribute to improved overall sleep quality. These effects could be due to mechanisms such as increased blood circulation, muscle relaxation, and modulation of ANS activity, which are particularly beneficial during high-intensity training phases.

2). Lines 418-423: This observation is likely related to competition stress and exercise-induced fatigue, which can have a stronger influence on sleep quality compared to the effects of cupping. These findings suggest that cupping may be more effective in improving sleep during relatively less stressful PSC period, where relaxation benefits can have a more pronounced effect, while in the ISC period, other factors such as stress and fatigue might overshadow the impact of cupping.

10. Strengths are well-highlighted, but limitations could be expanded. For example: Variability in individual responses to cupping; Potential placebo effects due to the nature of sham cupping.

**Response:** Thank you for your insightful feedback. In response, we have expanded the limitations section to address individual variability in response to cupping and the potential placebo effects associated with the intervention (in Lines 433-437). We believe these additions provide a more comprehensive discussion of the study's constraints.

**Changes in the manuscript**:

Fourth, individual variability in response to cupping may have influenced the results, as factors such as physiological differences and recovery capacity can vary among athletes. Fifth, due to the nature of sham cupping, potential placebo effects cannot be entirely ruled out, which may have contributed to subjective perceptions of improvement.

11. Practical recommendations for athletes and coaches are useful. Suggest areas for further research, such as cupping’s effects on recovery metrics in other sports.

**Response:** Thank you for the insightful feedback. In response, we have expanded the limitations section to include practical recommendations for athletes and coaches, as well as suggestions for future research on cupping’s effects across different sports and recovery metrics (Lines 440-446). We believe these additions will enhance the study’s applicability and provide valuable directions for further exploration.

**Changes in the manuscript**:

Practical recommendations include integrating cupping as part of a broader recovery strategy tailored to individual athlete needs and monitoring subjective and objective responses over time. Future research should focus on investigating cupping’s effects on specific recovery metrics, such as muscle fatigue, heart rate variability, and performance outcomes in athletes from different sports disciplines. Expanding the scope to include long-term interventions and different competitive levels may provide further insights into the practical applications of cupping in sports settings

**Conclusion**

12. The conclusion succinctly summarizes findings but could be more forward-looking, emphasizing the potential for refining cupping protocols.

**Response:** Thank you for the suggestion. I have updated the conclusion to highlight the potential for refining cupping protocols, including optimal frequency duration and cupping pressure, to maximize benefits for athletes (lines 454-456). I hope these revisions address the comment effectively.

**Changes in the manuscript**:

Future research could focus on refining cupping protocols, including optimal frequency, duration and cupping pressure, and extending studies to other athlete populations.

13. Suggest extending studies to other athlete populations or exploring long-term effects.

**Response:** Thank you for your valuable feedback. In response, I have revised the conclusion to not only highlight the potential for refining cupping protocols but also to suggest extending studies to other athlete populations and exploring the long-term effects of cupping (lines 456-458). These additions aim to provide a broader perspective on the future direction of this research.

**Changes in the manuscript**:

……and extending studies to other athlete populations. Additionally, exploring the long-term effects of cupping could provide further insights into its potential benefits. Overall, this study highlights the potential of cupping as a recovery strategy in baseball players.

**Reviewer: 3:** Your article contains all the essential data required for the topic, presented in a comprehensive and well-structured manner. The content is relevant and supports the objectives of the study effectively. I appreciate the clarity and depth of your analysis.

**Response:** Thank you for your kind and encouraging feedback. We are pleased to hear that the data presented in the article are comprehensive and well-structured, and that the content effectively supports the study's objectives. Your appreciation of the clarity and depth of our analysis is greatly valued, and we are grateful for your thoughtful review.

**Reviewer: 4:**

1. On pg 7, line 121, recommend you change 8 weeks to 4 weeks PSC and 4 weeks ISC, or 8 weeks total. The way it is currently written with "for 8 weeks" could be interpreted as 8 weeks consecutively.

**Response:** Thank you for your helpful suggestion. We have revised the text on page 7, lines 134-135, to clarify the duration of the study. It now reads as "4 weeks PSC and 4 weeks ISC", to avoid any confusion and ensure accurate interpretation of the study duration. We appreciate your attention to detail.

**Changes in the manuscript**:

Cupping was performed twice a week, with an interval of 2 to 3 days, for 4 weeks PSC and 4 weeks ISC.

2. On pg 7, lines 136-138, recommend changing the sentence to, "This questionnaire comprises a main question, which acquires data for classifying respondents’ shoulder joint function into three categories: easy, moderately difficult, and difficult."

**Response:** Thank you for your constructive suggestion. We have revised the sentence on page 8, lines 150-152, and we believe this change enhances clarity.

**Changes in the manuscript**:

This questionnaire comprises a main question, which acquires data for classifying respondents’ shoulder joint function into three categories: easy, moderately difficult, and difficult.

3. On pg 8, line 152, recommend changing "exceeded 1.1" to "exceeded 1:1".

**Response:** Thank you for your thoughtful suggestion. Upon review, we believe the original phrasing "respiratory exchange ratio exceeded 1.1" is correct. The respiratory exchange ratio (RER) typically exceeds 1.1 during high-intensity exercise, indicating an increased reliance on carbohydrates for energy. Conversely, at rest, the RER is generally around 0.7, reflecting a predominance of fat oxidation. Therefore, no changes were made to this part of the manuscript. We appreciate your attention to detail.

4. On pg 9, line 173, recommend considering explaining what "fast Fourier transform" is and possibly why it is used.

**Response:** Thank you for your insightful suggestion. We have added a brief explanation of "fast Fourier transform" (FFT) on page 9, lines 186-190 of the revised version. We believe this addition enhances the clarity and understanding of the analysis method used in the study.

**Addition in the manuscript**:

FFT is a mathematical algorithm that converts time-domain signals into frequency-domain signals, enabling the analysis of the power spectrum of HRV data. This method is commonly used in HRV analysis to assess ANS activity, as it helps differentiate between sympathetic and parasympathetic influences.

5. On pg 9, line 184, recommend removing "as follows:" and replace with "seven indicators:".

**Response:** Thank you for your helpful suggestion. We have revised the text on page 10, line 208, by removing "as follows:" and replacing it with "seven indicators:" to improve clarity and readability.

6. On pg 10, line 197, is the ISC period weeks 4-8 (or 5 weeks) or 5-8 (4 weeks)? If it is 4-8, then the ISC period is longer than the PSC period and week 4 is an overlap week. Will need to check data to make sure it matches the correct weeks.

**Response:** We greatly appreciate your insightful comment. Upon reviewing the study timeline, we would like to clarify that baseline data for the PSC period were recorded on Day 1 of week 1, prior to the initiation of the cupping intervention. Cupping was administered twice a week, typically on Tuesdays and Fridays, and continued until the end of Week 4, with the final recording on Day 27.

However, the baseline data for the ISC period were originally scheduled for Day 29 (Monday of Week 5) Given the close proximity of these two time points, and to avoid unnecessary repetition of measurements and disruption to the baseball team, the baseline data for ISC were instead recorded on Day 27. Therefore, both the PSC and ISC cupping interventions lasted for 4 weeks. The overlap in the fourth week was limited to Day 27, which marks the end of Week 4 and the beginning of week 5, rather than extending for a full 7 days.

We have confirmed the consistency of the data with this timeline. For further details, please refer to the revised **Figure 1**, which provides a detailed overview of the study timeline.

**Revised Figure 1:**

7. On pg 10, line 212; pg 13, line 255; AND pg 15, line 280, recommend "4-week cupping" or "cupping for 4 weeks"

**Response:** We appreciate the reviewer’s recommendation for consistency in phrasing. Following your suggestion, we have revised the text on pg 14, line 272, and pg 15, line 299 to consistently use the term "cupping for 4 weeks" instead of other variations. This adjustment ensures clarity and uniformity throughout the manuscript.

8. On pg 10, lines 207-217, can these two paragraphs be combined for PSC and ISC when there is no significant difference in data? Then, just focus on where there was a difference.

**Response:** We appreciate your thoughtful comment on improving the presentation of our findings. After reviewing this paragraph, we recognize that combining the paragraphs for PSC and ISC where no significant differences were observed could enhance clarity and streamline the narrative.

The revised section could emphasize the consistency of findings across both phases, noting the lack of significant changes or interactions. This change (lines 231–234 of the revised version) reduces redundancy and allows the discussion to focus more on areas with significant differences. We consider this revision to improve the manuscript's clarity and readability.

**Changes in the manuscript**:

After 4 weeks of cupping during both PSC and ISC, no significant changes were noted in the participants’ DASH questionnaire scores for upper-extremity disability and exercise disability or their FLEX-SF questionnaire scores (time effect). Additionally, no significant differences were observed between the groups, nor in the group × time interaction effect during either period (Table 1).

9. On page 11, line 221, need a period after the word "period"

**Response:** We appreciate your attention to detail. The period has now been added after the word "period" on page 11, line 238 of the revised version, to ensure grammatical correctness. Thank you for bringing this to our attention.

10. On pg 12, line 238, recommend removal of the word "only"

**Response:** We agree with your suggestion. The word “only” on page 12, line 255 of the revised version was deleted to improve the clarity and accuracy of the sentence. Thank you for highlighting this refinement.

11. On pg 14, Table 3, the HRV and Week(Trial) columns and the horizontal labeling is difficult to read or follow

**Response:** We appreciate your suggestion and have reformatted Table 3 to enhance its clarity and readability. The updated table has been presented in a landscape orientation, which better accommodates the data structure. To maintain the flow of the main manuscript, the reformatted table has been uploaded separately as supplementary information. We hope this addresses your concern.

12. On pg 17, line 315, recommend capitalizing "Zone Theory" to match other titles; same with line 318 "activation of the immune system theory"

**Response:** We appreciate your observation regarding title consistency. As suggested, "Zone Theory" on line 334 of the revised version and "Activation of the Immune System Theory" on line 337 have been capitalized to ensure consistency with other titles throughout the manuscript. Thank you for highlighting this improvement.

13. On pg 17, lines 315-318- are there references for these theories?

**Response:** These theories mentioned in the manuscript are cited from reference [33], which is a review article that compiles multiple studies and proposes various theoretical mechanisms to explain the effects of cupping therapy. This review consolidates the current understanding of the mechanisms behind cupping, citing relevant literature that supports each theory.

The reference for these theories can be found in this manuscript under reference [33].

14. On pg 19, lines 381-385, It is written that traveling the sleep quality improves (line 383) and then you state that travel negatively impacts sleep (line 384). This leads to confusion- recommend clarifying this point.

**Response:** Thank you for your valuable feedback regarding the potential contradiction in this article. Upon further review, we agree that the phrasing could lead to misinterpretation. We have clarified this section to ensure consistency and accuracy.

The results of Mah et al. (2018) (line 399 of the revised version) did not indicate that travel inherently improves sleep quality. Instead, the study found that athletes' sleep quality was worse on campus compared to when traveling for competitions. This suggests that the improvement in sleep quality during travel may be attributed to the reduction in the stress of high-intensity training on campus that requires waking up early, rather than travel itself.

Additionally, we addressed the negative impact of travel on sleep quality (lines 404-405) by highlighting specific factors such as jet lag, which is known to affect athletes' sleep during travel.

To enhance clarity, we have rewritten the text on pages 20, lines 398–406. We believe these changes address the reviewer’s concern and clarify the findings. Thank you for your suggestion, which has improved the manuscript.

**Changes in the manuscript**:

Notably, total PSQI score was consistently higher during PSC than during ISC. This finding is consistent with Mah et al. (2018), who reported that college athletes frequently have poor sleep quality, which tends to be worse on campus than during travel for competitions [35]. Although review articles have suggested that training, travel, and competition are the primary contributors to sleep disturbances in elite athletes, the main travel-related factor affecting sleep is jet lag [36]. However, the competitions held during the ISC period in this study did not involve jet lag, potentially minimizing its impact on sleep quality.

15. On pg 19, line 402, There is no previous mention in the Methods section that the data collection timeline was different per group, recommend clarifying this in the previous sections.

**Response:** Thank you for your insightful comment. We appreciate your attention to detail. In response, we have revised **Figure 1** in the Methods section to explicitly highlight the data collection timeline for each group, ensuring that this information is clearly presented. We believe these revisions will provide a clearer understanding of the study design and timeline.

**Revised Figure 1**:
